# Time-resolved nanosecond optical pyrometry of the vapor to plasma transitions in exploding bridgewires

**DOI:** 10.1038/s41598-021-86584-6

**Published:** 2021-04-02

**Authors:** T. A. Feagin, E. M. Heatwole, P. J. Rae, R. C. Rettinger, G. R. Parker

**Affiliations:** grid.148313.c 0000 0004 0428 3079 M-6, Los Alamos National Laboratory, MS-P917, PO Box 1663, Los Alamos, NM 87545 USA

**Keywords:** Engineering, Physics

## Abstract

Electrically exploded wires find uses throughout high-energy physics. For example, they are commonly used as high-temperature sources, X-ray generators, and in precision timing detonators. However, the detailed and complete physics that occurs is complex and still poorly understood. A full mechanistic description of these complex phenomena is beyond the scope of a single paper. Instead, we focus on the formation of metal vapor and its transition to plasma. This single transition is commonly assumed to comprise “bridge-burst”. We use a suite of diagnostics including a novel, fiber-based, high-speed, optical pyrometer to better characterize this transition. The primary finding from this project is that peak light output from an exploding wire does not temporally match the peak temperature. Additionally, it is found that peak light does not align with peak bridge-burst voltage and that the peak temperature is not voltage-dependent. These findings are non-intuitive and will allow for the correction of false assumptions previously made about this topic.

## Introduction

The measurement of thin, electrically-exploded metallic wires (bridgewires) has important applications including wire-array Z-pinch^1^, nanoparticle preparation^2,3^ and exploding bridgewire (EBW) detonators^4^. Power outputs of several MW into small samples of material can easily be achieved using modest capacitor discharge units (CDUs) making this technique attractive for bench-top, high-energy physics experiments. What is relatively unstudied is how this power is partitioned as it is transferred to the environment (e.g., heat, shock, etc.) and how changes to the initial energy supply affect this. The present understanding is that the bridgewire is superheated by the passage of a high current (typically hundreds of amps) and this results in rapid melting, vaporization, and ultimately formation of an expanding plasma shell and an associated shock process. Much of the early work examining larger wires and slower timescales can be found in the comprehensive review by Bennett^5^, while much of the more recent work on $\upmu$m wire and ns timescales can be summarized in selected works by Sarkisov, and Liu^6–10^.

The literature on this topic makes it apparent that the physics of bridge-burst is complex and generally poorly understood. A full review of this broad topic is therefore beyond the scope of this paper. Instead, this research primarily focuses on the point in the process where the wire material vaporizes and transitions to a plasma. This transition is important, as it is where previous researchers have identified the peak temperature and bridge-burst process occurs. Several models have been proposed to describe the processes of bridge-burst and to define the vapor-to-plasma transition^11,12^; however, most lack proper temperature parameters, relying instead upon absolute light intensity to estimate temperatures. To our knowledge, no research has been done to determine directly-measured temperatures of exploding wires on the nanosecond time scale or how they correspond to the voltage, current, energy, and light intensity.

Researchers have used multi-color optical pyrometry to make high-speed measurements of temperatures in other high-energy physics applications such as explosives, shocked materials, and fireballs^13–18^. The recent designs reviewed by Ota et al.^17^ provide compelling examples of the strength of multi-color pyrometry for time-resolved nanosecond temperature measurements in dynamic environments. However, much of the work found in the literature focuses on shocked solid acceptor materials heated to temperatures below, or just above, melting. The measurement and characterization of isolated exploding wires using optical pyrometry appears not to have been undertaken. What is more, the majority of the pyrometers found in the literature are designed to only function in evacuated cells behind specially coated LiF lenses. When challenged with ionizable gases present in a real atmosphere, the reliability of these systems typically suffers greatly owing to the presence of intense spectral lines, particularly at extreme temperatures (>5000 K). The pyrometer developed for this work specifically addresses the limitations of other designs (spectral lines, signal to noise, speed, etc.) using a combination of interchangeable narrow bandpass filters to alleviate spectral line influence, selectable electrical loads to optimize the temporal resolution of the measurements, and novel noise reduction and error propagation methods to maximize the usable signal obtained. The design for the pyrometer is presented in extensive detail in the supplementary information.

In this paper, we use commercially available RP-80 gold bridgewire detonator headers (www.TeledyneRISI.com) as our exploding wire and spark gap generator (SGG) platforms. The advantage of using the RP-80 header is the high level of reproducibility available from a relatively inexpensive disposable device. Pairing these headers with a high-speed, multi-color optical pyrometer and custom CDUs enabled the simultaneous examination of the temperatures and intensities produced in exploding wires at a variety of voltages and input energies.

We hypothesized that what others have assigned as peak temperature during bridge-burst is incorrect and needed further research to improve models and guide future associated experimental work. We demonstrate that for exploding wires, the magnitude of the burst intensity is generally not coupled to temperature and that the use of this common and intuitive assumption will result in large errors in the models that rely upon intensity-based temperature estimates. Additionally, in testing this hypothesis several interesting new features of bridge-burst were identified.

## Experimental

### Variable-voltage experiments

Variable-voltage experiments were carried out at discrete voltages from 500 to 2,000 V. RP-80 headers with and without gold bridgewires were tested and analyzed in this range. These tests were performed using a general purpose CDU comprised of a 5 $\upmu$F high-voltage capacitor (PN 775D505980-104, www.sbelectronics.com) triggered by an optical S38 HV switch (PN S38-FO, www.siliconpower.com). The S38 switch is capable of 3–4 ns timing jitter, if driven correctly, and can operate between 500–4,000 V. The output current was measured with an integral 20 MHz Pearson current-viewing transformer (model 5046, www.pearsonelectronics.com) and the voltage across the copper lead wires was measured by a 50 MHz bandwidth CalTest CT4079-NA floating high-voltage probe. Owing to the inductance of these lead wires, the voltage measured at this location differs from that across just the gold wire, but practically it is extremely difficult to make an accurate measurement inside the header. Instead we measured the inductance of the detonator lead loop (*L*) and the measured voltage was corrected to a bridge voltage using the voltage across an inductor $V=-L\ di/dt$. The signals were digitized on a Tektronix MSO58LP 12 bit oscilloscope at 1.25 GS/s. Time-resolved optical pyrometry was acquired for all tests.

### Variable gas environment experiments

A custom, small, pressure cell was built that allowed for the insertion and precise alignment of an RP-80 header with the pyrometer optic. This cell was used to determine the effect different environmental conditions had on the bridge-burst process. The burst process was examined at 1 & 5 atm of air and 1 & 5 atm SF$_6$. As with the experiments in “Variable-voltage experiments” section, the total intensity of light output, temperature, and electrical voltage and current were all measured. All experiments were performed at a charge voltage of 2,000 V using the general use CDU described above.

### Conduction time experiments

Variable-timing current-cut-off (conduction time) experiments were performed using a CDU comprised of a 5 $\upmu$F capacitor in series with a fast SiC MOSFET switch and the EBW. This allowed the current duration to be altered, thereby decoupling the total energy input into the system from the energy required for just the wire to burst. The on-off switch used was a Cree model CAS120M12BM2 with a CGD15HB62 driver circuit capable of holding off 1.2 kV (www.wolfspeed.com). The maximum transient current rating is greater than 480 A and has a rated on-to-off time of 70 ns when driving a low inductance circuit. To avoid over-driving the switch voltage at burst time, the CDU was only charged to 600 V and an extra flywheel diode was placed across the switch in case the output was under-damped. With this large capacitance, low-inductance, and 600 V, the CDU was still capable of exploding the RP-80 headers. Time-resolved optical pyrometry measurements were acquired as for the variable-voltage experiments.

### Total light energy emission experiments

In order to measure the total light emission energy from the wire heating processes a Thorlabs ES120C pyroelectric energy sensor was used. This is a slow sensor that integrates the total light energy from 185 nm to 25 $\upmu$m incident on the sensor into a calibrated peak voltage signal output over approximately 20 ms. The signal is therefore an exponentially rising and then relatively slowly exponentially decaying signal with different time constants where the peak value reached is the total absorbed energy measurement. Pyroelectric materials are also piezoelectric and so the air shock associated with the wire burst was found to produce a rapid sinusoidal oscillating output from the detector that contaminates the signal. The peak was therefore extracted by fitting a double exponential form to the sensor output voltage to effectively smooth the rapid oscillation and recover the valid underlying measurement. This approach was deemed preferable to placing a transparent baffle between the exploding wire and the sensor to suppress the air shock pressure since the effect to the sensor calibration factor with a baffle in place would not be known over the entire wavelength range.

## Results and discussion

As an initial proof of concept for the pyrometer described above, we measured plasma temperatures of air-arcs using a spark gap generator (SGG) made from an RP-80 detonator header lacking a bridgewire. Prior research on SGG and the temperatures produced enabled validation of our pyrometer^19–22^. The methods used in the literature from Bye et al. required averaging over multiple strikes; this is a luxury not afforded to those working with thin metal bridges. However, it is useful to compare maximum intensity, temperature and duration between our measurements and those found in the literature. The maximum temperatures ($\approx$ 14,000 K) measured with the optical pyrometer used throughout this work indeed align well with those found throughout the literature that used similar energy sources. Additionally, as expected, the temperature of the air-arc is relatively long-lasting compared with the wire explosion event and tracks the current (i.e., the peak temperature of the air-arc occurs at the peak current).

**Figure 1:**
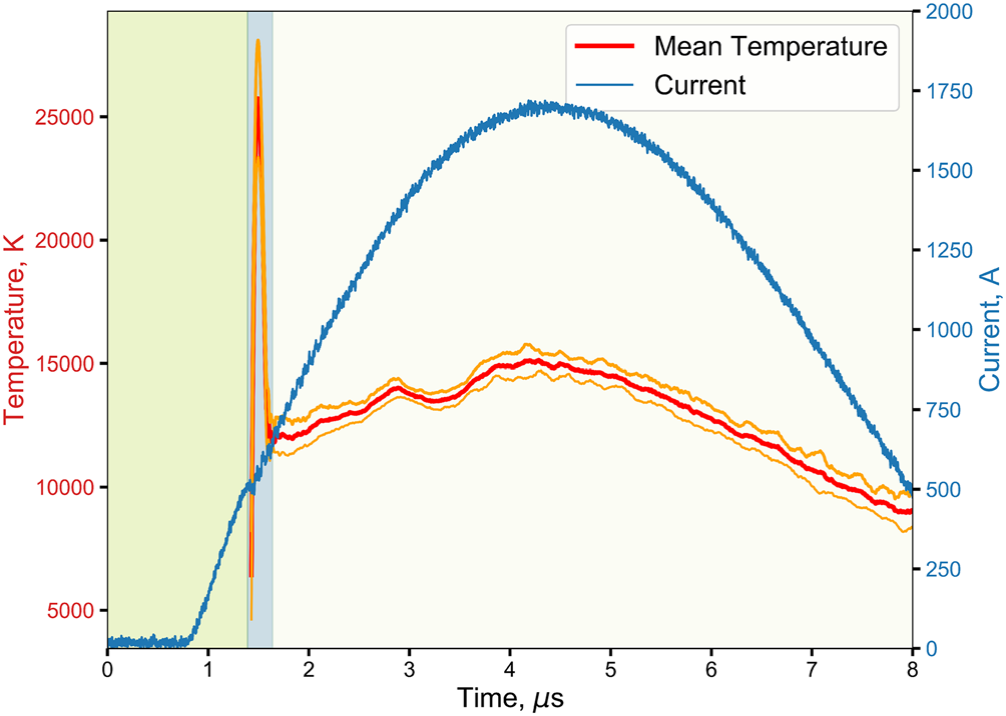
Temperature and current profiles of a bridge-burst at 2 kV. Shaded regions represent wire heating/melting and vaporization (green), plasma (blue), and air-arc (off-white). Bridge-burst actually occurs at the transition from the green to blue region just before the slight current dip and temperature increase.

The bridged headers consist of a 38 $\upmu$m diameter by 1 mm long gold bridgewire, spot welded to copper terminals. Figure 1 shows the temperature and current trace of the bursting of an RP-80 gold bridge. The bridge-burst trace can be divided into three distinct regions: (1) the initial rise in current and melting of the bridge; (2) a sharp, short duration ($\approx$ 100 ns) temperature increase due to the actual bursting of the bridge; (3) a restrike and the formation of an air-arc that looks identical in temperature and intensity to that of the SGG used in our proof of concept experiments. It is apparent from Fig. 1 that peak current no longer represents peak temperature although the temperature in the air-arc region does track the current. This finding is important to defining what “bridge-burst” means as we explore the temperature and intensity profiles of bursting bridges throughout this paper. As such, we define bridge-burst as the transition from the green to the blue region in Fig. 1.

### Variable-voltage

**Figure 2:**
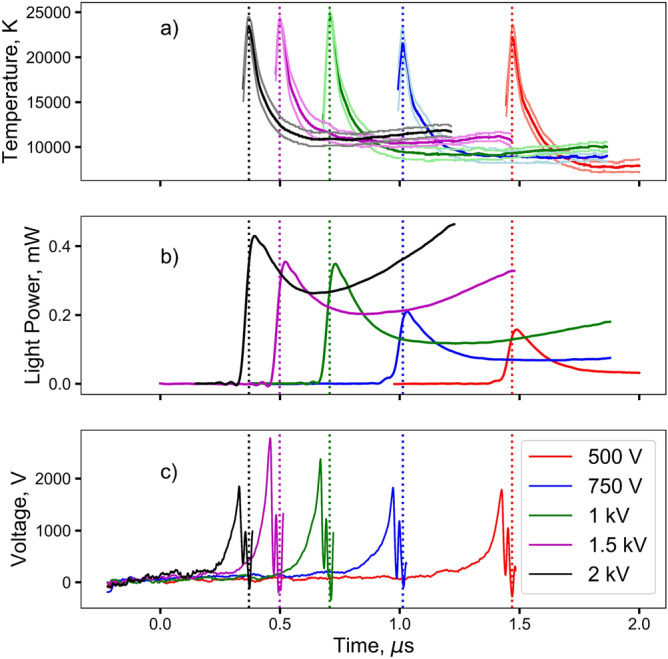
Results from the variable-voltage series. (**a**) Temperature versus time for various CDU voltages. (**b**) Incident light power for 445–455 nm. (**c**) The corresponding voltage versus time traces. The dotted lines indicate time of peak temperature. For clarity only the bridge-burst is shown and the subsequent long duration arc section has been cropped.

To understand how peak temperature correlates to peak intensity, we first examined the voltage dependency of the bridge-burst using the variable-voltage CDU. The experimental voltage series used for this work ranged from 500 to 2,000 V. Figure 2 presents an overview of the experimental results for the test series, where the time-dependent temperature and light output power of the bursting wires are shown. It is seen that the increased voltage leads to a greater current derivative which in turn leads to higher electrical powers with a corresponding increase in electrical energy deposited before bridge-burst. The observed decrease in time-to-burst with increasing voltage coupled with increased energy deposition and burst-action (the time integral of current squared) prior to burst is an established result, although it conflicts with the common assumption that burst-action is constant for a given wire material and geometry^23^.

Figure 2 also reveals the unexpected result that the peak temperature of the bridge-burst is not dependent on the voltage applied to the CDU. That is, despite more electrical energy being deposited into the wire at higher voltages, the measurements show peak temperatures similar to the lower voltage tests. However, the intensity of light emitted during bridge-burst is dependent on the voltage, as intuition would suggest. The implementation of a one-way ANOVA test indicates that the whole voltage series does not reach the same peak temperature; however, the Student T-test indicates that the 1, 1.5, and 2 kV tests come to a statistically indistinguishable temperature, which suggests the temperature asymptotes with increasing voltage ^24^. Further, it is observed that the peak temperature does not quite align with the slightly delayed (50–60 ns) peak light intensity. This new finding differs from the prevalent assumption commonly used in the literature and may result in significant difference in some circumstances.

When considering just the bridge-burst process which appears to be decoupled from, and unsupported by, the subsequent air-arc, it is hypothesized that the misalignment of peaks is due to the fact that at peak temperature the plasma ball from the bridge-burst has not yet expanded enough to fill the 1 mm aperture of the pyrometer optic, and is in effect self-shielding its emission. As the plasma region expands, it cools radiatively as a result of having performed work on the surroundings. However, the expanding plasma is both taking up more of the area of the optic and is physically closer to the optic, leading to more light being collected despite the cooler temperatures measured. While this explains why the peak temperature appears before the peak light intensity, it was initially unclear why the light intensity at peak temperature is higher for higher voltages while the temperature remains constant.

**Figure 3:**
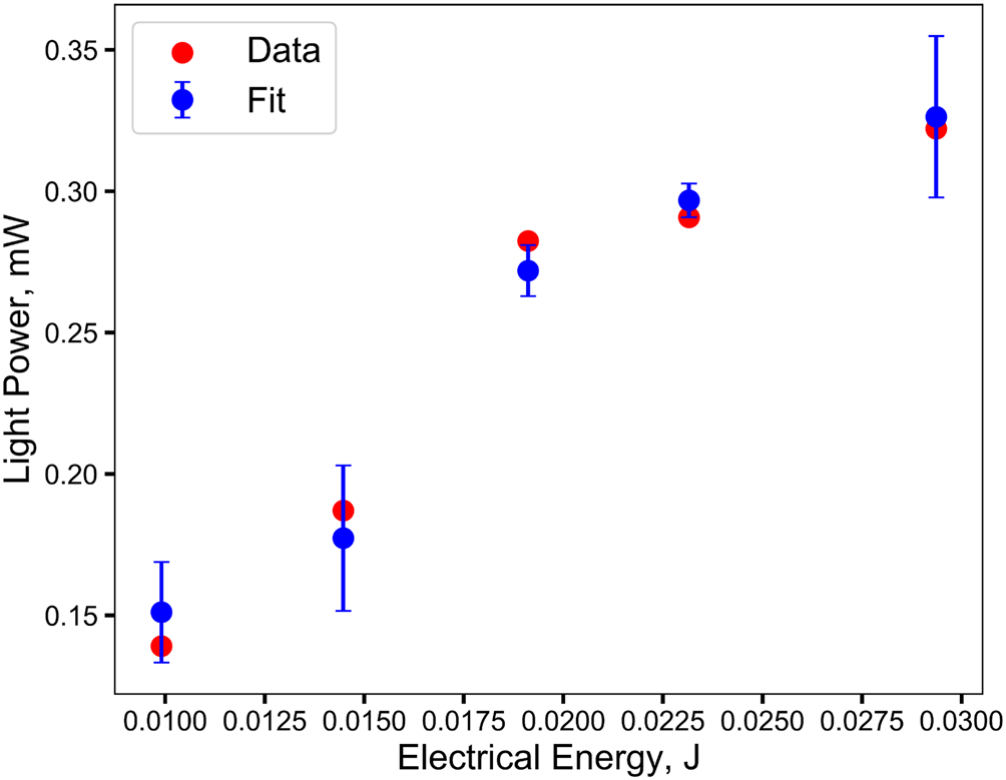
The fit of Eq. 1 to the light power and electrical energy versus the experimental data. Error bars on the fit come from the corresponding observed temperature ranges.

Previous research examining air-arcs provides clues to why this may be the case^25–28^. Olsen and others have observed a temperature ceiling, where an increase in current no longer leads to an increase in temperature, but rather the diameter of the arc increases with increasing current. This would explain the observation that while the light power grows as the voltage is increased, the temperature remains essentially constant. If it is assumed that the arc reaches a constant temperature and any additional energy contributes to widening the diameter of the arc, it is expected that the emitted radiant energy will be proportional to the surface area of the arc times the spectral energy predicted by Planck’s law. Since the energy required to heat the arc will increase proportionally to the cube of the radius, it is expected that the emitted spectral energy will increase as follows:

1 $$\begin{aligned} E_l = \left( aE_v^{\frac{2}{3}} + b\right) \int _{\lambda _1}^{\lambda _2} B\left( \lambda , T\right) d\lambda \end{aligned}$$

where $E_l$ and $E_v$ are the light and electrical energy and *a* and *b* are the fit constants. To obtain the electrical energies the electrical power from the peak of the voltage spike to the temperature peak is integrated and Eq. 1 is then fitted to the measured light powers. Figure 3 shows good agreement between the observed data and the hypothesis.

To test these theories, further experiments under variable environments were performed to determine whether the gold wires functioned similarly to the air-arcs described by Olsen.

### Variable environment

The short-lived, high-temperature spike seen in the variable-voltage experiments above poses two significant questions: what is the mechanism of formation and why does it appear to behave similarly to an air-arc (i.e., the temperature asymptotes with increasing voltage and current but the total light output continues to increase)? We hypothesized that this temperature spike was due to the electrical breakdown of air around the gold wire as it bursts.

During the electrical breakdown of air, free electrons are accelerated by the electric field until they impact another molecule. If the kinetic energy of the electron is larger than the ionization energy of the molecule, another electron is freed and both electrons are accelerated by the electric field, whereby the process repeats, leading to a chain reaction and the formation of an arc. For this process to occur there needs to be a large enough mean free path between collisions for the electron to gain a sufficient amount of kinetic energy to ionize the impacted molecule. Therefore, if an air-arc is a contributing factor in the production of the observed short-lived, high-temperature spike, then presumably it can be suppressed by increasing the ionization energy of the atmosphere (using SF$_6$), or by decreasing the length of the free path between the molecules (i.e., by increasing the air pressure).

**Figure 4:**
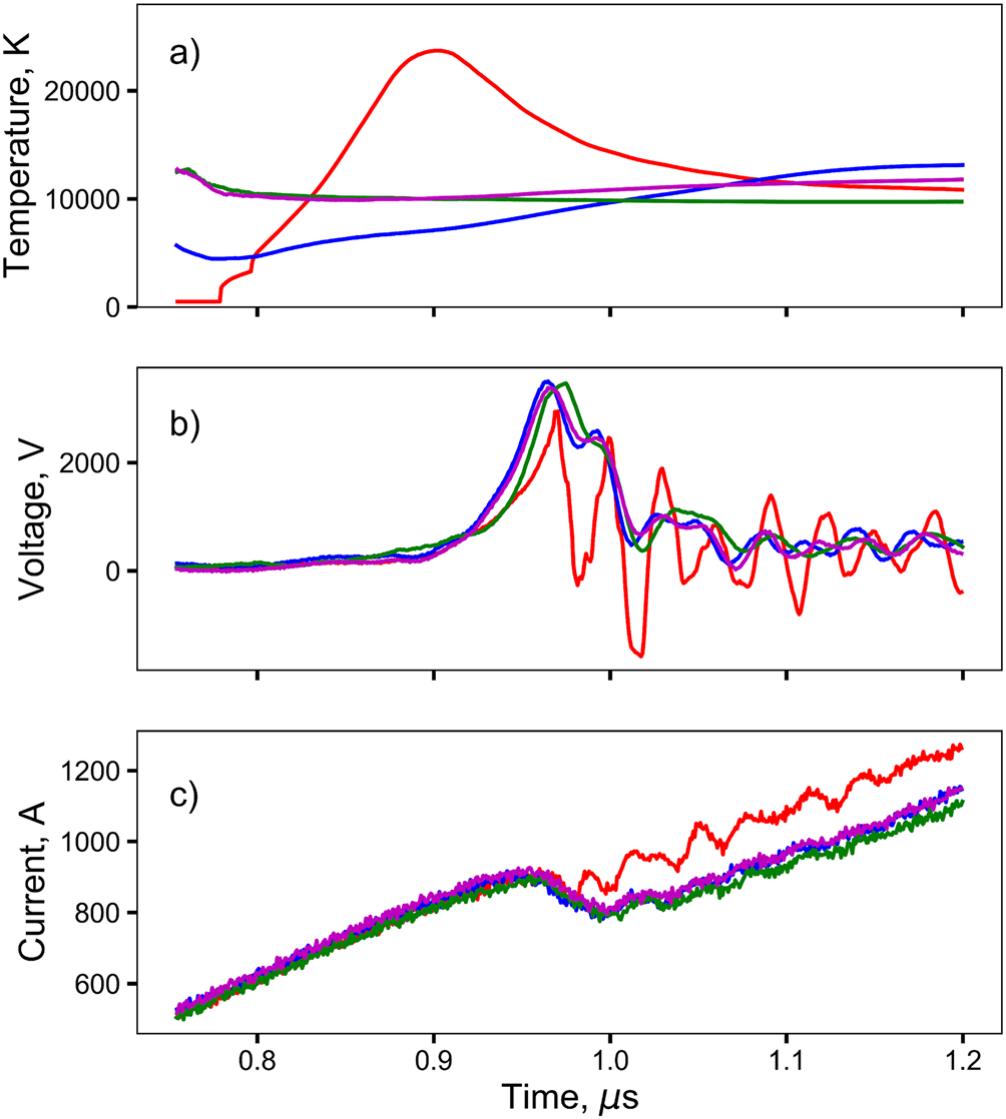
Temperature, voltage and current profiles of bridge-bursts at 2 kV in air and SF$_6$. The initial temperature spike is suppressed in the 5 atm air and SF$_6$ environments. Red(1 atm Air), Blue(5 atm Air), Green(1 atm SF$_6$), Purple(5 atm SF$_6$).

Figure 4 compares the temperature spike in the initial 1 atm air experiments presented previously with the new data. It is seen that the peak temperature is suppressed in 1 & 5 atm of SF$_6$ and 5 atm of air. Additionally, the voltage traces in the 1 & 5 atm of SF$_6$ and 5 atm of air lack the very rapid decrease during the burst event seen in the 1 atm of air. This, combined with the decrease in overall current seen in the 1 & 5 atm of SF$_6$ and 5 atm of air during the bursting processes suggests that in regular bursts in 1 atm of air the electrical breakdown of air produces a low-resistance current shunt path in parallel to the conducting wire burst process. This increases the total current but reduces the potential across the wire terminals owing to the finite impedance of the CDU and cabling. This finding is similar to that previously published for long wires burst in vacuum, but where the rapid liberation of high vapor pressure material in the wire creates an ionizable medium surrounding the wire, see for example ^9,10^.

Further, temperatures in the 1 & 5 atm of SF$_6$ and 5 atm of air tests are significantly lower (10,000–15,000 K) than the temperatures observed in the 1 atm of air experiments (24,000 K). It is therefore clear that the air-arc process at 1 atm produces greybody emissions at significantly greater temperatures than the gold wire burst process. During the initial very rapid heating of the wire, it has been proposed that the thermodynamic path follows the spinodal for the material and results in the formation of a super-critical fluid and heterogeneous vaporization^10,12,29^. Such a process would limit the superheated vaporization temperature to approximately 7800K for gold rather than the equilibrium boiling temperature of approximately 3100K^10^. However, once the gold vapor is formed a conduction path is created that allows ionization and the formation of a heavy ion plasma. It is presumed that the temperature of such a plasma is lower than the corresponding light ion plasma reported earlier.

### Variable conduction time

To further understand the results from the variable-voltage experiments, we examined the effect of providing 600 V to the wire and then cutting off the current at prescribed times during the burst process using a custom built on-off CDU. By altering the conduction time we could identify a distinct threshold between bridges that explode rapidly and violently and ones that did not. In this context an explosion is defined as a sharp spike in temperature and light intensity that results from a rapid vapor expansion creating an expanding plasma shell and associated electrical conduction processes.

It was determined that if the variable conduction time CDU was allowed to deliver current for 1 $\upmu$s or longer, the wire would burst and all data collected would look similar to that of the general purpose CDU at 600 V. As such, 1 $\upmu$s was chosen as our “hard burst” criteria. By incrementally reducing the conduction time it was found that the explosion threshold was $\approx$ 700 ns (a “soft burst”). Four representative time points were therefore chosen to cover the range (700, 750, 775, and 900 ns).

**Figure 5:**
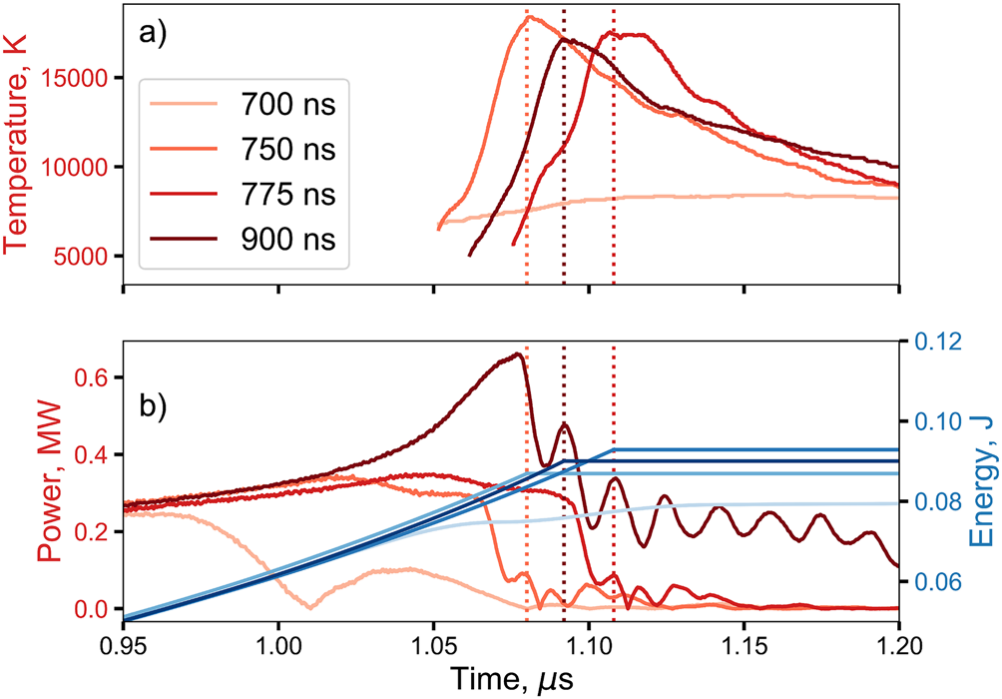
Results for a series of conduction time experiments. (**a**) Temperature versus time for various conduction durations. (**b**) The corresponding electrical power versus time where the dotted vertical lines correspond to peak temperature for the bridge-burst in 1 atm of air presented in (**a**).

As Fig. 5 shows, for this CDU at fixed voltage, the electrical energy deposited in the wire until peak temperature does not depend on conduction time, even though the total electrical power supplied during the whole process has dropped by approximately a factor of four for the shorter conduction times compared with the longest one. The burst-action until peak temperature was calculated and found to be consistent across all conduction times where bridge-burst occurred, which was expected for an on-off CDU charged to a fixed voltage. This contrasts with the previous variable-voltage CDU series, where both the electrical energy and action increased with increasing voltage. As with the variable-voltage CDU, the data from the variable conduction time CDU provides further confirmation of the surprising finding that peak temperature does not align with peak emission intensity. More intuitively, the peak temperature does not appear to be strongly dependent on the conduction time if explosive bridge-burst occurs.

### Total light energy emission

**Figure 6:**
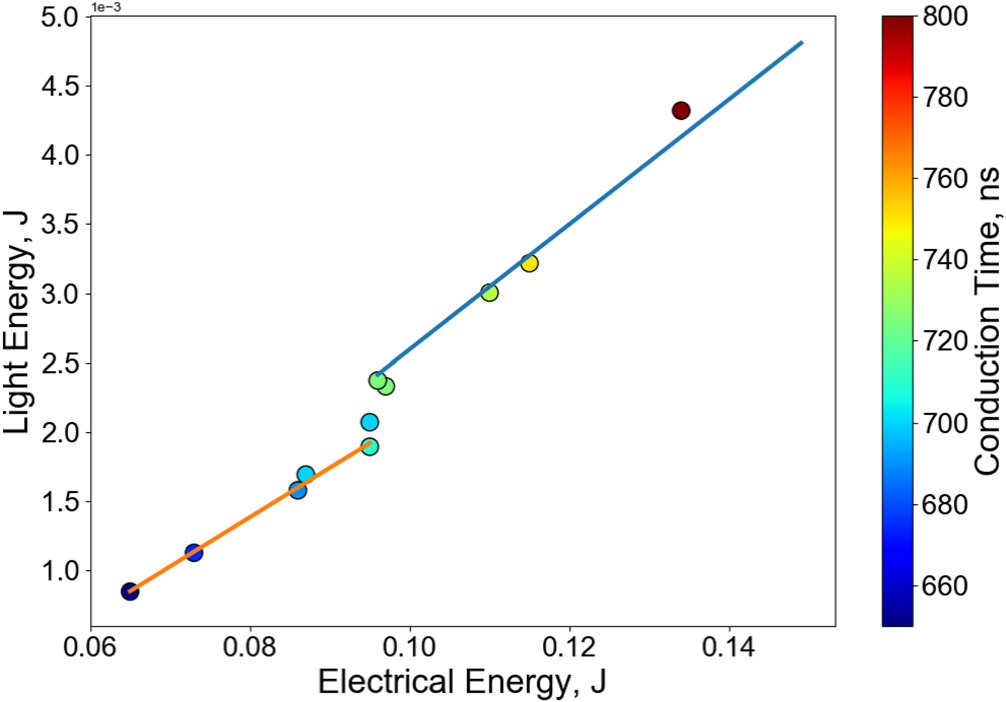
Plot of total light energy output versus electrical energy input for a series of conduction time tests at 600 V. The color of the circle indicates the conduction time. The blue line represents conditions where a violent wire explosion process was observed, while the orange line represents conditions where it was not.

To better quantify the variable conduction time results, the total light emission energy versus conduction time was measured using the identical RP-80 headers. Figure 6 summarizes a plot of total light emission energy from wavelengths of 185 nm to 25 $\upmu$m versus the total electrical energy deposited in the wire for a range of conduction times from significantly below the duration required for a hard burst (650 ns) to significantly above (800 ns). The feature which stands out is the jump in light energy at $\approx$ 0.095 J of electrical energy input corresponding to a conduction of time of $\approx$ 700 ns. This time was previously identified as producing a violent bridge-burst event and a high temperature spike.

It is proposed that this sudden jump in light energy corresponds to conditions where the heating of the wire is sustained for sufficient duration that the resulting increased wire resistance begins to reduce the overall current flow. The system inductance then leads to an increase in potential across the bridge terminals to counteract this reduction in current. The resulting voltage spike and the simultaneous onset of vaporization in the bridge-wire create conditions that ionize the surrounding air and result in a parallel (and very bright) air-arc conduction process in addition to the underlying violent wire burst process.

However, at conduction times of less than $\approx$ 700 ns an incomplete and slow vaporization process occurs in the heated bridgewire that does not result in a rapid violent explosion or accompanying air-arc, but it does still emit lower intensity light over a longer period.

## Conclusions

This investigation into the physics involved in the vapor explosion and resulting generation of an expanding plasma shell of exploding wires led to three new findings. Firstly, it was shown that the common assumption that peak intensity also defines peak temperature for a bridge-burst is incorrect and needs revision. Since the peak temperature and intensity are dependent upon the specific bridge-wire material, geometry and burst environment, the experiments and diagnostics developed within this paper are intended to enable others to more easily quantify parameters for their specific systems.

Secondly, it was discovered that peak temperature was not dependent on the applied voltage for the CDU, cable and bridge-wire system used. Instead, it was found that as the voltage applied to the CDU was increased, the intensity of light increased while the peak temperatures remained stable over a broad range of CDU charge voltages. This is a non-intuitive result; however, the emission wavelength ratios appeared to remain Planckian for all voltages.

Thirdly, it was identified that there was a critical point in the wire heating process where conditions were created that resulted in an air-arc occurring in parallel to the underlying wire burst process. If electrical heating is suspended before this point then a weak, low temperature, slow burst process occurred exclusively in the wire. However, heating for a longer duration resulted in a violent explosive event and the formation of a very brief high-temperature air-arc followed by a lower temperature sustained plasma conduction process.

## Supplementary Information

Supplementary Information.
