# Supplementary material for: Time-resolved nanosecond optical pyrometry of the vapor to plasma transitions in exploding bridgewires
Source: Sci Rep. 2021 Apr 2;11:7467. doi: 10.1038/s41598-021-86584-6 (PMC8018943; doi:10.1038/s41598-021-86584-6)
Supplement: Supplementary file 1 — Supplementary Information. [file 41598_2021_86584_MOESM1_ESM.pdf]

Time-Resolved Nanosecond Optical Pyrometry of the Vapor to Plasma Transitions in Exploding Bridgewires

T A Feagin, E M Heatwole, P J Rae, R C Rettinger, and G R Parker
Corresponding Author: Trevor A Feagin Feagin@lanl.gov
M-6, Los Alamos National Laboratory,
PO Box 1663, MS-P917,
Los Alamos, NM 87545, USA

January 14, 2021

1 Supplementary Information

1.1 Pyrometer

Optical pyrometry is a method through which temperature measurements can be acquired by fitting the spectral radiance of a target to a Plank blackbody,

$$B(\lambda, T) = \frac{2hc^2}{\lambda^5} \frac{(d\lambda dA dS_r)}{e^{\frac{hc}{k_B \lambda T}} - 1}, \tag{1}$$

where $B(\lambda, T)$ is the spectral radiance, dA is collection area, and dS_r is steradians. However, to accurately represent the temperature several other factors must be taken into consideration. For a single channel pyrometer, $I(t)$ can be used to determine temperature through the following,

$$I(t) = AS_rC \int_{\lambda_i}^{\lambda_f} F(\lambda)S(\lambda)\epsilon(\lambda)B_\lambda(\lambda, T)d\lambda \tag{2}$$

where, I is the current, A is the sample area, S_r is steradians, C is a calibration factor that accounts for losses from fiber optics, etc., λ_i and λ_f comprise the bandwidth range of the individual bandpass filter used, F(λ) and S(λ) are correction factors for the filter set and photodiode sensors used, respectively.

One of the primary contributors to the uncertainty present in pyrometry data arises from unknown and changing emissivities of samples. Unfortunately, the errors

introduced by changing emmisivities are not easily overcome using single channel wide band measurements, and as a result it is difficult to present meaningful temperature measurements with such instruments. However, for ratiometric pyrometry, especially when considering the temperatures of plasmas in exploding bridgewires, the error due to emissivity is expected to be minimized allowing for accurate temperature and errors to be calculated [?] [?]. By making the assumption that the emissivity of the plasmas measured are wavelength independent over the narrow ranges we have chosen, we can eliminate emissivity as a variable. Additionally, a big advantage of the ratiometric pyrometer approach is that the need for precise calibration of area and steradians is no longer necessary. This significantly reduces the errors introduced through the difficult distance dependent calibrations required of a single channel design. By using 4 colors, 6 ratiometric measurements can be acquired using the following,

$$\frac{I_1(t)}{I_2(t)} = \frac{C_1 \int_{\lambda_i}^{\lambda_f} F_1(\lambda) S_1(\lambda) B_\lambda(\lambda, T) d\lambda}{C_2 \int_{\lambda_i}^{\lambda_f} F_2(\lambda) S_2(\lambda) B_\lambda(\lambda, T) d\lambda} \tag{3}$$

To actually calculate the instantaneous temperature the ratios acquired during an experiment are numerically integrated over time.

1.2 Pyrometer design and setup

The two types of biased un-amplified detectors used for all of the experiments were silicon photodiodes (ThorLabs DET025AFC) with 150 ps rise times and InGaAs photodiodes (DET08CFC) with 110 ps rise times. The detectors were attached directly to a 1 GHz bandwidth 12-bit oscilloscope (Tektronics MSO58). Data were acquired at 6.25 GSamples per second but internally averaged to an effective sample rate of ≈1 GSamples per second to reduce noise.

The input of the pyrometer consisted of a fiber coupled optical element (ThorLabs TC06FC) attached to a 400 μm 0.39 NA fiber optic. This fiber was then tapered to 200 μm 0.22 NA using a custom drawn fiber taper (CeramOptec) to minimize optical losses. The taper was then split three ways using 200 μm 0.22 NA 1×2 fiber splitters (ThorLabs TM200R3F1B). Two 75:25 and one 50:50 splitter was used providing for a split ratio of 28 %, 28 %, 25 %, 19 % to be distributed to the four channels (Figure 1). Splitting the inputs asymmetrically allowed for more signal to be preferentially sent to the channels that typically had lower response in the temperature range of interest.

The split inputs were then independently filtered using narrow bandpass filters from Thorlabs (430 ± 10 nm, 450 ± 10 nm, 1064 ± 10 nm, 1200 ± 10 nm). This filter set was chosen specifically to avoid known spectral lines at both high and low temperatures, which otherwise would contribute greatly to measurement error in the system. The choice to use the narrow bandpass filters despite the resulting significant reduction in signal level was made to minimize the errors associated with differences

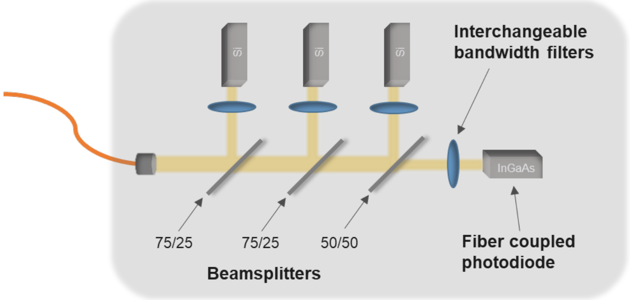

Figure 1: Design and setup for the multi color pyrometer.

in radiation intensity as a function of temperature over the individual measurement spectral window. That is, using narrow bandwidths minimizes the error that results from a change in spectral intensity gradient versus temperature within the pass band at two different wavelengths.

Additionally, by selecting two of the filters in the near UV and the remainder in the near IR, the simultaneous measurement of the extremely high temperatures expected as the bridge begins to burst (≈15,000 K) as well as the subsequent cooler (≈8,000 K) air arc seen after, could be made.

The introduction of the fiber splitters to the optical path leads to a slight change of path length to each detector. For very fast events, like a bridge-burst, the difference in path length needed to be corrected for in order for the instantaneous ratio between the wavelengths to be accurate.

The primary advantage to the all-fiber based pyrometer as opposed to the free air systems found throughout the literature is robustness and small form factor. Once built, these systems need none of the frequent adjustments or alignments that are necessary with free space designs. Additionally, the small form factor has allowed for 8 independent channels of the 4-color pyrometer to be incorporated into a single compact 3 inch rack mount system.

The un-amplified biased photodetectors used in this design produce an output current that is proportional to the incident light intensity over an extremely wide power level (hundreds of nanowatts to hundreds of milliwatts). The current is converted to a convenient measurement voltage by flowing through a current viewing resistor, the value of which can be chosen to optimize the measurement temporal resolution versus signal magnitude. The temporal resolution is limited by the requirement to charge and discharge the photodetector junction capacitance, any stray capacitance, and the input capacitance of the digitizer. This occurs primarily through the current viewing resistor. For all the temperature measurements presented here, a 1 kΩ load was selected with a time constant of 21.5 ns.

1.3 Pyrometer calibration and analysis

One difficulty with the temperature measurement of a bursting bridge wire is the lack of calibration sources of high enough temperatures. Specifically, there must be sufficient radiance in the shorter wavelengths for a calibration to be performed. In order to overcome this a halogen lamp (Stellernet SL1-Cal) of known spectrum was used. A correction for dark current and voltage offset error in the digitizer was performed. The signal from the lamp over the four wavelengths was measured with the identical setup as used in the experiment. However, even with an extremely bright calibration lamp, very little signal is received at the shorter wavelengths, making it necessary to average the data at the scope to remove some of the random noise. Additionally, the current viewing resistor value was increased from the 1 kΩ load, used for the experimental work, to 1 MΩ for calibration to raise the signal to noise level adequately. The linear response of the detectors allowed this to be done with no further corrections required.

What we are interested in is the current produced by the photodetector unmodified by the circuit, since it will be proportional to the incident light. Measurements at the oscilloscope can be approximated as the response of an RC circuit driven by the current from either the silicon or InGaAs photodetector. Therefore, the dynamic response of the RC circuit must be solved to obtain the time-resolved current. This requires that the time constant, $\tau = RC$, be known. The solution to this system is well known and can done by directly considering the ODE modeling the circuit:

$$I(t) = \frac{V(t)}{R} + C\frac{dV(t)}{dt} \tag{4}$$

Where $I(t)$ and $V(t)$ is the time-dependent current and measured voltage, while R and C are the resistance and capacitance of the system. The current can be easily found by calculating the time-derivative of the voltage signal and applying equation 4.

When this method is used to transform the voltage to current the data are extremely noisy. There are several sources for this noise including the high-frequency noise from the electronics, noise from the bridge-burst electrical process, and noise from the CDU function itself. The noise from the CDU and bridge-burst proved to be extremely difficult to isolate and required the use of digital filters to remove. This artifact must be removed from the signal since any time varying component not originally in the photodiode signal that is near or above the time constant of the RC circuit will be amplified by transforming from voltage to photocurrent. The lower frequency aspects of the CDU noise was removed by implementing a digital notch filter from Python's scipy.signal package. The signal was then smoothed using a second order Savitzky-Golay filter where the window length of the the filter was optimized by using an algorithm similar to the one developed by Sadeghl and Behnia [?]. The filtered and smoothed voltage data was then transformed as described previously to calculate the original instantaneous current produced by the sensor.

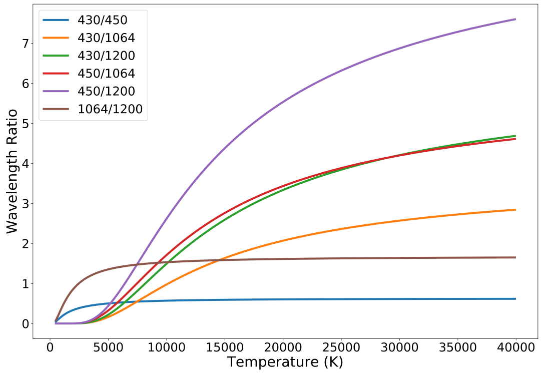

Figure 2: Showing the wavelength ratios without multiplication by the calibration constant for the wavelengths used in this experiment. Both the 430/450 nm and 1064/1200 nm ratios are extremely flat for temperatures greater than 5,000 K and are therefore unsuitable for these high temperatures.

In order to calculate the temperature the integrals in Equation 3 were numerically computed using the manufacturer supplied data for the specific optical filter and photodiode response. The unknown wavelength sensitivity was then calibrated by using the response to the Stellarnet SL1-Cal halogen light source to generate the calibration term for each wavelength ratio. This in-turn was then used to generate a temperature lookup table for each wavelength ratio. The interpolated lookup table was used to calculate the temperature for each relevant wavelength ratio at every time step of measured data.

Figure 2 shows the extrapolated wavelength ratios with respect to temperature before the use of the calibration factor. The wavelength ratios 430/450 nm and 1064/1200 nm are extremely flat for temperatures greater than 5,000 K, which means any noise or error in the measurement will lead to unacceptably large errors in the temperature measurement. Therefore, these ratios were discarded and the 430/1064, 430/1200, 450/1064, and 450/1200 nm ratios were used to determine the temperature in what follows.
